# Standardizing Periocular Surface Electromyography: A Scoping Review of Methods and Emerging Applications

**DOI:** 10.3390/jcm15062256

**Published:** 2026-03-16

**Authors:** Larysa Krajewska-Węglewicz, Ewa Filipiak, Małgorzata Dorobek

**Affiliations:** 1 Department of Ophthalmology, National Medical Institute of the Ministry of Interior and Administration, 02-507 Warsaw, Poland; 2 Department of Neurology, National Medical Institute of the Ministry of Interior and Administration, 02-507 Warsaw, Poland

**Keywords:** electromyography, orbicularis oculi muscle, eyelids, facial muscles, signal processing, review

## Abstract

**Background:** Surface electromyography (sEMG) of periocular muscles is a non-invasive technique used to assess eyelid dynamics and facial neuromuscular function, with applications in ophthalmology, neurology, and rehabilitation. Despite its clinical and research potential, substantial methodological variability—particularly in electrode placement, acquisition parameters, and signal processing—has limited reproducibility and hindered broader clinical translation. A comprehensive synthesis of existing methodologies was therefore needed to support future standardization. **Objectives:** The review aimed to systematically map current periocular sEMG methodologies, identify sources of methodological heterogeneity, organize findings into structured methodological domains, and develop a conceptual framework along with a minimum reporting set to promote transparency, reproducibility, and comparability across studies. **Eligibility Criteria:** Studies were eligible if they investigated surface electromyography of periocular muscles and reported methodological details related to electrode placement, signal acquisition, processing, or analysis. Randomized controlled trials, observational studies, and pilot investigations were included. No restrictions were placed on publication year. **Sources of Evidence:** Comprehensive searches were conducted in PubMed, Embase, and Web of Science from database inception through November 2025. Grey literature sources were also examined to enhance coverage and reduce publication bias. **Charting Methods:** Two reviewers independently screened records and extracted data. Extracted information was organized into predefined methodological domains. A thematic synthesis approach was used to identify recurring methodological patterns, and findings were integrated into a structured conceptual framework. **Results:** Sixteen studies published between 2002 and 2025 met the inclusion criteria, encompassing randomized trials, observational studies, and pilot investigations. Considerable heterogeneity was identified across studies in electrode characteristics, placement strategies, reference configurations, sampling frequencies, and normalization procedures. Three recurring methodological domains emerged: instrumentation and acquisition, analytical and normalization approaches, and clinical or experimental applications. Based on these domains, the authors developed a conceptual methodological framework and proposed a minimum reporting set intended to improve methodologyical transparency and support reproducibility and multicenter comparability. **Conclusions:** Periocular sEMG represents a promising yet methodologically fragmented field. This scoping review provides the first comprehensive synthesis of periocular sEMG practices and establishes an evidence-based platform for standardized acquisition, processing, and reporting. Adoption of the proposed framework may strengthen reproducibility, facilitate multicenter collaboration, and accelerate integration into clinical and research settings.

## 1. Introduction

Surface electromyography (sEMG) is a widely used technique for non-invasive assessment of muscle activity with high temporal resolution and established applications across neuroscience, rehabilitation, and biomedical engineering [1,2]. Methodological advances have primarily focused on limb and trunk muscles, supported by standardized protocols such as SENIAM [3]. In contrast, periocular sEMG has received comparatively limited methodological attention despite its relevance for quantifying eyelid function and facial neuromuscular control.

Dysfunction of periocular muscles contributes to conditions such as facial nerve palsy [4], blepharospasm [5], synkinesis [6], ptosis [7], lagophthalmos [8], and age-related eyelid laxity [9,10,11,12]. Reliable assessment is, therefore, clinically important for diagnosis, surgical planning, and postoperative evaluation. Beyond clinical use, periocular sEMG is increasingly incorporated into wearable technologies and human–computer interfaces [13]. However, methodological development has not kept pace with these expanding applications.

Recording from the orbicularis oculi (OOM) and levator palpebrae superioris (LPS) presents distinct challenges, including limited electrode placement area, anatomical complexity, susceptibility to crosstalk, and uncertainty surrounding normalization strategies when maximal voluntary contraction (MVC) is impractical [14,15,16]. Consequently, heterogeneous acquisition protocols and analytical workflows hinder reproducibility and cross-study comparison.

Despite growing interest, the methodological landscape of periocular sEMG has not been systematically consolidated. A scoping review is, therefore, appropriate to map existing approaches, identify technical gaps, and clarify priorities for standardization.

The objective of this review is to support methodological transparency and future standardization of periocular sEMG, thereby facilitating reproducible research, multicenter collaboration, and progression toward clinical and technological applications.

## 2. Materials and Methods

### 2.1. Protocol and Registration

This scoping review was conducted in accordance with the Preferred Reporting Items for Systematic Reviews and Meta-Analyses extension for Scoping Reviews (PRISMA-ScR) [17] and the Joanna Briggs Institute (JBI) methodological framework [18]. The protocol was prospectively developed following the PRISMA-P structure. A completed PRISMA-ScR checklist has been submitted as Appendix A to ensure compliance and transparency.

### 2.2. Eligibility Criteria

Eligibility criteria were defined using the Population–Concept–Context (PCC) framework recommended for scoping reviews.

- Population: Human participants undergoing sEMG recording of periocular muscles, including the OOM and LPS.
- Concept: Use of sEMG to assess muscle function, dysfunction, or treatment effects related to eyelid movement in clinical, surgical, rehabilitative, or experimental settings.
- Context: Studies from any discipline (e.g., ophthalmology, neurology, rehabilitation, biomedical engineering) reporting periocular sEMG outcomes.

Eligible study designs included randomized controlled trials (RCTs), observational studies, technical or methodological reports, case series, relevant conference abstracts, doctoral theses, and registered but unpublished trial protocols.

Exclusion criteria comprised animal or cadaveric studies, investigations using intramuscular EMG exclusively, purely computational models without empirical data, and studies focusing solely on non-eyelid facial muscles.

### 2.3. Information Sources and Search Strategy

A comprehensive and reproducible search strategy was developed in consultation with an experienced medical librarian. Searches were performed in PubMed (MEDLINE), Embase (Elsevier), and Web of Science (Clarivate) from database inception to November 2025, without language or publication year restrictions. All search databases and applied limits are fully reported in the manuscript.

The search strategy combined controlled vocabulary (MeSH and EMTREE terms) with free-text keywords related to electromyography and periocular anatomy. Core controlled terms included Electromyography, Orbicularis Oculi Muscle, and Eyelids, supplemented by keywords such as “surface electromyography”, “sEMG”, “eyelid muscle”, “orbicularis oculi”, and “levator palpebrae superioris”. Boolean operators, truncation, and proximity functions were optimized for sensitivity and precision.

The full PubMed search strategy and database-specific adaptations for Embase and Web of Science are provided in Appendix B, ensuring full reproducibility of the literature search in accordance with PRISMA-ScR recommendations.

Grey literature was searched via OpenGrey, ProQuest Dissertations & Theses Global, and clinical trial registries (ClinicalTrials.gov, WHO International Clinical Trials Registry Platform). Conference proceedings from major ophthalmology, neurology, and biomedical engineering societies (ARVO, ISCEV, IEEE EMBC) were screened through Embase and Web of Science Conference Proceedings. Additionally, reference lists of all included articles and related reviews were hand-searched to identify additional eligible studies.

### 2.4. Selection of Sources of Evidence

Two reviewers (L.K.-W. and M.D.) independently performed title and abstract screening using predefined eligibility criteria. Full-text articles of potentially relevant records were then retrieved and assessed independently by the same reviewers. Screening was conducted using structured spreadsheets specifically developed for this review to ensure consistency and traceability of decisions.

Discrepancies at both the title/abstract and full-text screening stages were resolved through discussion. When consensus could not be reached, disagreements were adjudicated by a third reviewer (E.F.). All inclusion and exclusion decisions were documented, and reasons for exclusion at the full-text stage were recorded.

### 2.5. Data Charting Process and Synthesis

A standardized data charting form was developed a priori in Microsoft Excel following JBI guidance and piloted on three randomly selected studies to refine clarity and consistency. Extracted domains included bibliographic information, study design, population characteristics, muscles investigated, electrode configuration, acquisition parameters, analytical and normalization methods, primary outcomes, and reported limitations.

Inter-reviewer agreement during pilot testing reached Cohen’s kappa = 0.86, indicating strong consistency.

Studies were iteratively grouped to identify recurring methodological patterns and gaps across three domains: (1) instrumentation and signal acquisition, (2) analytical and normalization strategies, and (3) clinical and experimental applications. Comparative mapping to facial and limb sEMG literature helped contextualize periocular-specific constraints, including limited recording surface and anatomical overlap.

Findings were integrated into a conceptual methodological framework encompassing electrode configuration, acquisition, signal processing, analysis, and reporting.

### 2.6. Methodological Considerations

In line with scoping review conventions, a formal risk-of-bias assessment was not performed [18]. However, methodological limitations identified during extraction—including small sample sizes, non-standardized electrode placement, and incomplete reporting of acquisition parameters—were systematically recorded to contextualize results and guide recommendations for future studies.

## 3. Results

### 3.1. Search Results and Study Characteristics

**Figure 1:**
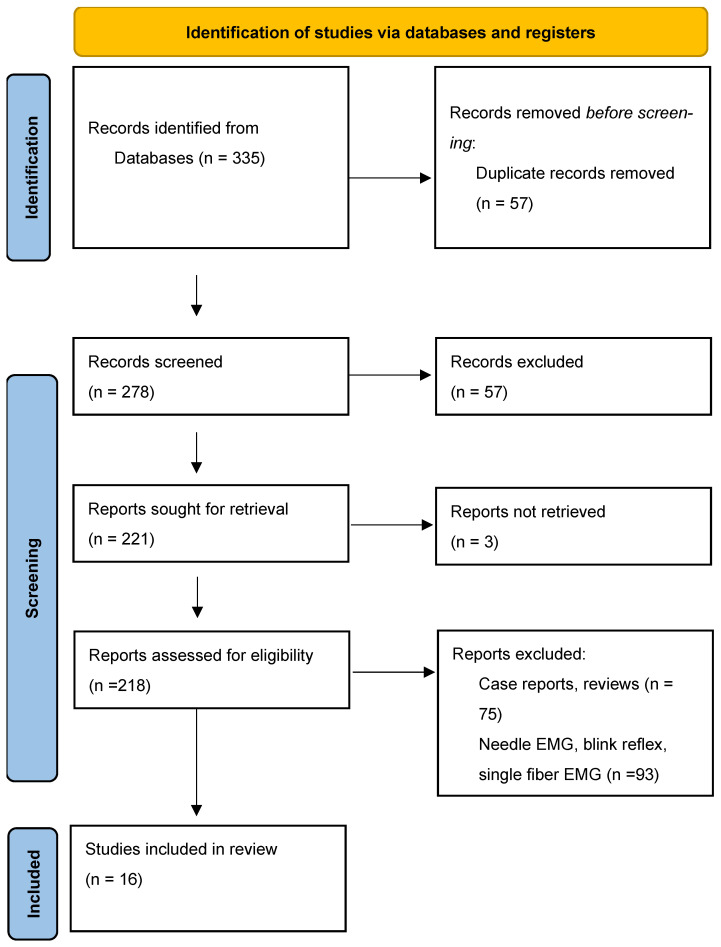
Distribution of included studies by year of publication. Source: Page MJ, et al. *BMJ* **2021**, *372*, n71. https://doi.org/10.1136/bmj.n71 [32].

Database searches across PubMed, Scopus, Embase, and Web of Science retrieved 335 results and unique records. After duplicate removal and full-text screening, 16 studies published between 2002 and 2025 met the inclusion criteria (Figure 1).

**Figure 2:**
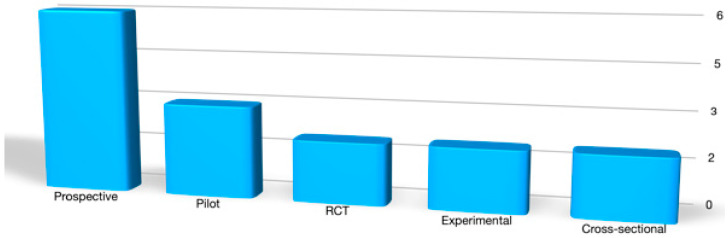
Types of study designs identified in eyelid sEMG research.

Included designs comprised two randomized controlled trials [19,20], six prospective studies [7,21,22,23,24,25], three pilot or feasibility studies [14,26,27], two methodological or experimental investigations [15,28], two cross-sectional studies [29,30], and one longitudinal observational study [31] (Figure 2).

**Figure 3:**
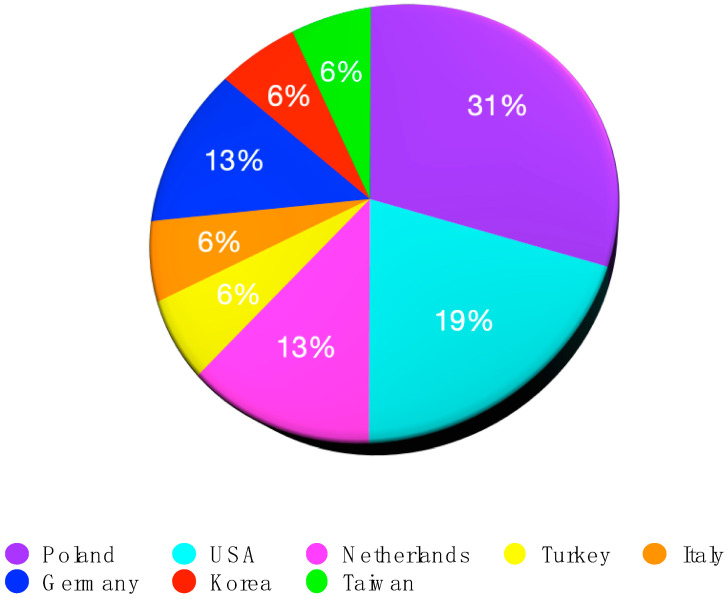
Distribution of studies by country of publication.

Combined, these studies represented 560 participants (range 5–84) across eight countries, most frequently the United States [21,27,28], Poland [7,14,25,29,30], and The Netherlands [20,31] (Figure 3).

Thirteen studies examined the OOM exclusively, while three also evaluated the LPS [24] or frontalis activity [20,23]. The included literature spans more than two decades, during which sEMG technology evolved markedly from analog bipolar Ag/AgCl electrodes with 10–450 Hz bandwidths in early 2000s studies to digital amplifiers, ≥2000 Hz sampling, and miniaturized or high-density arrays post-2018 [27]. These innovations improved spatial resolution and signal fidelity but also amplified methodological heterogeneity, complicating direct comparison across time periods. Nevertheless, recent publications show convergence toward standardized placements, digital acquisition, and task-specific normalization. Recent studies demonstrate partial convergence toward standardized electrode placement, digital acquisition, and normalization procedures; however, consensus regarding montage configuration, reference location, and scaling strategy remains lacking.

### 3.2. Population and Study Context

Participant populations were diverse, encompassing both healthy volunteers and patients with neurological, age-related, or post-surgical eyelid disorders. Normative cohorts demonstrated that OOM activity remains consistent across sexes and between eyes, supporting the methodological assumption of bilateral equivalence in periocular sEMG research [29]. Age, however, showed a significant negative association with maximal amplitude, mean amplitude, and RMS during maximal voluntary contraction, indicating an age-related decline in neuromuscular performance [30].

Six studies investigated neurological dysfunctions—particularly facial nerve palsy and hemifacial spasm—to monitor recovery [31], evaluate botulinum toxin efficacy [19], or support biofeedback rehabilitation for synkinesis [7]. Five addressed surgical or aesthetic contexts, such as blepharoplasty [20], ptosis repair [7,24], or forehead rejuvenation [20]. Three explored physiological mechanisms of blinking in healthy individuals [15,28,29].

The broad range of designs (RCTs versus observational) influenced comparability: controlled interventional studies typically standardized stimuli or pharmacologic exposures (e.g., botulinum toxin), while exploratory or pilot studies optimized electrode placement or signal analysis. As a result, findings across these contexts are not directly comparable, and evidence supporting specific methodological choices is often context-dependent. Table 1 lists the included studies evaluating the basic features.

Table 2 provides a structured overview of electrode configuration, acquisition parameters, analytical outcomes, and limitations across the included periocular sEMG studies, underscoring substantial methodological variability and the need for standardized recording practices.

A structured summary of methodological constraints identified across the included studies is presented in Table 3. Common constraints included small sample sizes, heterogeneous designs, and incomplete reporting of key acquisition parameters, often limiting reproducibility. Although reporting improved in more recent investigations, substantial methodological variability persists.

### 3.3. Methodological Synthesis

#### 3.3.1. Instrumentation and Acquisition Variability

**Figure 4:**
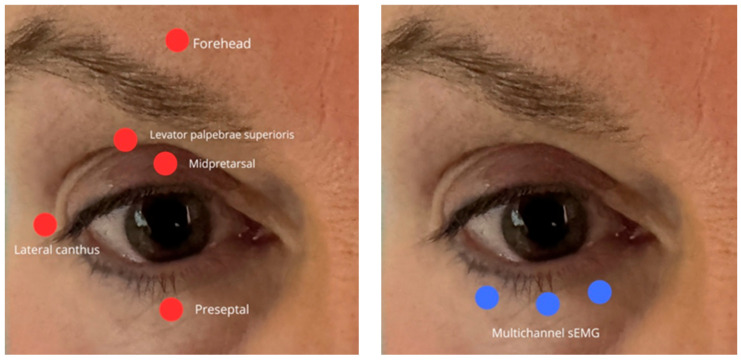
Electrode placement sites for sEMG recordings in periocular muscles.

Marked heterogeneity existed in electrode type, placement, inter-electrode distance, and reference position (Figure 4). Two dominant configurations were identified: pretarsal montages, targeting the mid-pretarsal OOM for clinical or post-surgical assessment, and lateral canthal or mixed montages, applied in experimental and blink-modulation paradigms. Inter-electrode distances ranged from 10 to 20 mm, while reference electrodes were variably placed on the forehead, mastoid, or cheek. No consensus was observed regarding an optimal reference site, and several studies did not justify their reference choice.

Only a minority of reports, such as Schneider et al. [23], provided schematic maps and fixed spatial coordinates, meeting reproducibility standards.

Most studies used Ag/AgCl disposable electrodes [7,15,24,26,27,28,29,30] and commercial amplifiers (Delsys, Biopac, Nihon Kohden) with gains of 1000–2000× and 10–500 Hz bandwidths. Sampling frequencies between 500 and 2000 Hz were adequate for capturing OOM activation. While notch filtering at 50/60 Hz and full-wave rectification were common, normalization procedures varied substantially, and several studies omitted normalization entirely, limiting inter-study and inter-subject comparability.

This variability mirrors early limb sEMG literature before the adoption of SENIAM guidelines, indicating that periocular sEMG remains at a pre-standardization stage.

#### 3.3.2. Analytical Approaches and Normalization

Amplitude-based metrics such as root mean square (RMS), mean absolute value (MAV), and peak amplitude dominated outcomes in thirteen studies [7,14,15,19,21,22,23,24,26,27,28,30,31], while temporal analyses of latency, duration, or blink frequency were used in three [15,28,29]. Four reports assessed inter-side symmetry, especially in unilateral conditions [7,19,22,31]. Only a minority employed within-subject scaling [30] or adaptive normalization [7]. Spectral power analysis and nonlinear EMG parameters were not applied in the included studies. Recent normative work reinforced normalization to maximal voluntary contraction (%MVC) as a strategy to reduce interindividual variability and improve clinical interpretability [29]. Normative datasets reinforced their value for establishing reference intervals, while ratio-based metrics (e.g., OOM/LPS) demonstrated sensitivity to pathological imbalance and postoperative neuromuscular recalibration [7].

Analytical strategies clustered into two approaches: amplitude-focused pipelines quantifying treatment-related change, and temporal analyses targeting blink physiology.

Direct comparisons between these methods were absent, and no study systematically evaluated the impact of normalization on outcome interpretation.

#### 3.3.3. Temporal Epoch Definition and Blink-Related Signal Interpretation

Temporal epoch definition was inconsistently reported despite its importance for physiological interpretation. Blink-related studies typically synchronized EMG analysis with blink onset and offset using kinematic or electrophysiological triggers, enabling separation of baseline activity, peak contraction, and relaxation phases.

In contrast, clinical investigations predominantly employed fixed or task-based epochs during sustained closure, voluntary blinking, or resting gaze. These designs capture overall muscle activation but may obscure discrete blink dynamics.

No standardized approach to epoch duration, segmentation, or spontaneous blink handling was identified. As a result, variability in reported outcomes likely reflects analytical methodology as much as underlying physiology. Explicit reporting of epoch definitions is therefore critical for reproducibility and cross-study comparison.

#### 3.3.4. Clinical and Translational Applications

Periocular sEMG was applied across three primary domains: diagnostic monitoring of neuromuscular disorders (facial palsy, synkinesis, blepharospasm) [19,23,26], surgical outcome evaluation (blepharoplasty, ptosis, rejuvenation) [7,22,24], and experimental validation or device innovation (EOG integration, AI-guided adaptive stimulation) [15,26,27,28,29].

Task-specific recording during voluntary closure, blinking, or facial expression was common to nearly all studies. More recent research expanded this scope to include emotion- or attention-modulated tasks [7] and AI-adaptive feedback systems [26], demonstrating the growing methodological sophistication and translational potential of eyelid sEMG. The emergence of normative activation benchmarks and coordination-based biomarkers further supports the clinical interpretability of periocular EMG signals [12].

Nevertheless, small sample sizes and heterogeneous protocols continue to limit definitive conclusions regarding clinical superiority or translational readiness.

#### 3.3.5. Temporal and Technological Evolution

Given the long observation window (2002–2025), technological progression constitutes an independent dimension of heterogeneity. Early studies using analog amplifiers and larger electrodes yielded lower signal-to-noise ratios, whereas contemporary research employed digital, miniaturized, and in some cases high-density sEMG arrays [24]. This technological evolution introduced both opportunities for improved signal quality and additional sources of methodological inconsistency, particularly in electrode density and data processing pipelines.

### 3.4. Summary of Key Insights

Despite persistent variability, recent investigations indicate a gradual movement toward methodological consistency, particularly in electrode placement and normalization practices. Normative datasets further suggest a shift from exploratory applications toward quantitatively anchored periocular sEMG.

However, agreement on core methodological parameters has not yet been achieved. The field appears positioned for standardization but remains short of universally accepted best-practice guidance.

## 4. Discussion

### 4.1. Overview

This scoping review represents the first systematic mapping of methodological approaches in periocular sEMG, spanning more than two decades of research. Periocular sEMG lies at the intersection of clinical neurophysiology, ophthalmology, rehabilitation, and biomedical engineering. Across 23 years of publications, we identified consistent growth in applications—from neurology and oculoplastics to reconstructive surgery and bioengineering—yet reproducibility remains hindered by heterogeneity in electrode configuration, acquisition parameters, and data analysis.

From both clinical and technological perspectives, standardized methods are essential to enable reproducible data collection, integration with high-density and wearable systems, and interoperability with multimodal biosignal platforms (e.g., electro-oculography, photoplethysmography, inertial sensors). The principal contribution of this review is, therefore, methodological rather than clinical; it clarifies how periocular sEMG is currently implemented and identifies priority areas for standardization.

Across studies, inconsistent alignment between analysis epochs and blink physiology emerged as a central limitation. Because blinking represents the primary functional output of the OOM, failure to synchronize recordings with blink dynamics risks conflating tonic activation with phasic neuromuscular events.

Such variability reflects a lack of consensus guidelines tailored to the periocular region rather than shortcomings of individual investigations. Blink-synchronized approaches are most common in neurophysiological research targeting reflexive and voluntary blinking, whereas fixed-duration epochs are typically used in clinical, surgical, and rehabilitative contexts where overall activation, symmetry, or therapeutic response is the primary concern.

Recent normative and prospective observational work signals a gradual shift toward quantitatively interpretable metrics. Age-adjusted activation patterns and coordination-based indices, such as ratios reflecting LPS–OOM interplay, suggest that periocular sEMG is evolving from descriptive measurement toward functional neuromuscular modeling. Such developments may ultimately support physiologically grounded reference standards.

The methodological constraints identified across the included studies should be considered when interpreting the findings of this review. Frequent limitations—such as small sample sizes, heterogeneous study designs, inconsistent electrode placement, and incomplete reporting of acquisition and normalization procedures—reduce comparability and restrict the strength of methodological inferences. Consequently, observed differences in periocular sEMG outcomes may reflect variation in recording practices rather than true physiological divergence. While the synthesis enables identification of recurring methodological patterns, the evidence base remains insufficiently standardized to support definitive technical recommendations. These considerations reinforce the need for cautious interpretation and underscore the importance of developing consensus-driven recording frameworks. Importantly, these limitations do not diminish the relevance of periocular sEMG but rather highlight the field’s transitional stage between exploratory investigation and methodological consolidation.

#### 4.1.1. Impact of Study Design on Data Comparability

Study design exerted a measurable influence on the interpretability of periocular sEMG data. Interventional designs offer improved control of confounding variables but often lack standardized acquisition timing relative to treatment effects, particularly when neuromuscular responses evolve over weeks. Conversely, observational studies contributed valuable innovations in placement mapping and processing pipelines, yet frequently introduce variability in participant state, task execution, and recording conditions. Normative datasets provide an essential methodological anchor by establishing reference intervals against which pathological patterns can be evaluated. At the same time, demographic influences—particularly age-related reductions in activation—highlight the need for stratified reference values when comparing heterogeneous cohorts.

Prospective observational approaches appear especially informative for capturing dynamic neuromuscular changes, emphasizing that design selection should align with the intended physiological inference. Harmonization of acquisition timing, electrode placement, and task definitions will be critical to improving cross-study comparability and enabling future meta-analytic synthesis

#### 4.1.2. Comparison with Existing sEMG Frameworks and Rationale for a Periocular-Specific Model

Methodological standardization in sEMG has advanced significantly in limb [33,34] and trunk muscles [35,36] through initiatives such as the SENIAM guidelines, and comparable consensus efforts have been made for facial muscles like the frontalis [37], zygomaticus [38], and masseter [39,40]. These frameworks improved reproducibility by defining electrode placement, filtering, and normalization protocols.

However, periocular musculature presents unique anatomical and functional constraints that preclude direct adoption of existing standards. The OOM and LPS are small, densely layered muscles characterized by rapid activation dynamics and proximity to adjacent facial and extraocular muscles, increasing susceptibility to crosstalk and motion artifacts [21,40]. Building on—not replacing—established sEMG principles, our synthesis adapts these frameworks to the periocular context through anatomy-aware considerations, including electrode miniaturization and pretarsal alignment, filtering strategies to mitigate ocular and frontal interference, and normalization approaches suitable for rapid, low-amplitude activations. These adaptations form the conceptual basis of a periocular-specific framework rather than a finalized standard.

The absence of a reliable maximal voluntary contraction (MVC) protocol remains a central methodological barrier in periocular sEMG, necessitating alternative normalization strategies. Several approaches may support inter-subject comparability when true maximal effort is impractical. Within-subject scaling, in which signals are normalized to each participant’s peak activation during a standardized task, can reduce interindividual variability and is particularly suited to longitudinal or interventional designs. Adaptive normalization relative to baseline muscle activity may further enhance sensitivity to subtle neuromuscular changes, provided that baseline recordings are stable and artifact-free. Task-based normalization, including scaling to spontaneous or standardized blinking, offers a functionally anchored reference aligned with the primary physiological role of the orbicularis oculi. Complementary strategies—such as submaximal reference tasks (e.g., gentle or forced eyelid closure), ratio-based metrics reflecting LPS–OOM coordination, and emerging normative datasets enabling reference-based interpretation—may further improve analytical consistency.

Although none of these methods has achieved consensus, systematic evaluation and prioritization of normalization frameworks should be considered essential for advancing methodological standardization, improving inter-study comparability, and supporting future clinical translation of periocular sEMG.

#### 4.1.3. Derivation of the Methodological Framework

The proposed framework was developed inductively through thematic synthesis of study design, acquisition, analysis, and reporting practices. 

Each included study was mapped to five domains—design, population, acquisition, analysis, and outcomes—and recurrent patterns were distilled into three interdependent pillars governing data quality and interpretability.

1. Instrumentation consistency: electrode geometry, reference configuration, and sampling/filtering parameters.
2. Analytical and normalization clarity: alignment of amplitude- versus time-based metrics with study objectives.
3. Contextual alignment: ensuring that diagnostic, surgical, or experimental goals dictate task and timing choices.

These elements form a structured workflow—from electrode configuration to reporting—that is intended as a guide for methodological transparency rather than a prescriptive protocol. Table 4 operationalizes this framework into a concise reporting set designed to support reproducibility.

### 4.2. Practical Recommendations: A Minimum Reporting Set for Periocular sEMG

To accelerate harmonization, we propose a concise minimum reporting set for periocular sEMG studies.

1. Anatomy/placement:

Precise montage (pretarsal or lateral canthal), inter-electrode distance (mm), reference site, and photographic or schematic documentation.

Example: electrodes positioned approximately 4–6 mm above the superior eyelid margin along the midvertical meridian, with an inter-electrode distance of ~10 mm and a ground electrode placed on the forehead (e.g., Fp1 according to the international 10–20 system).

2. Acquisition:

Electrode type, amplifier model, gain, bandwidth, sampling rate, notch filter settings, and skin preparation.

Example: bipolar Ag/AgCl electrodes connected to a differential amplifier (gain 1000–2000×), band-pass filtered at 20–500 Hz, sampled at ≥1000 Hz, with skin cleansed using alcohol to reduce impedance.

3. Tasks/state:

Clear task definitions and participant conditions, including timing relative to interventions.

Example: three repetitions of gentle eyelid closure followed by forced closure, each lasting 3–5 s with standardized rest intervals.

4. Preprocessing/analysis:

Rectification, envelope filtering, artifact handling, normalization strategy, and primary analytical metrics.

Example: full-wave rectification followed by RMS extraction using a 50-ms moving window, with signals normalized to peak activation obtained during standardized voluntary closure.

5. Data transparency:

Primary outcomes with dispersion measures, missing-data handling, adverse events, and availability of processed datasets where feasible.

Example: reporting RMS values as mean ± SD with predefined exclusion criteria for motion artifacts.

### 4.3. Technological Evolution and Temporal Heterogeneity

Technological progress has improved signal fidelity but simultaneously expanded methodological diversity. Early analog systems were constrained by low sampling rates and larger electrodes, whereas contemporary platforms employ digital amplification, miniaturized sensors, and advanced preprocessing algorithms.

Innovation typically precedes standardization; accordingly, future guidance should distinguish technology-invariant principles from device-specific refinements to maintain compatibility across evolving hardware ecosystems.

### 4.4. Clinical and Engineering Implications

Methodological standardization is a prerequisite for clinical translation. Reliable periocular sEMG could enable objective monitoring of neuromuscular recovery, early detection of dysfunction, and quantitative evaluation of surgical outcomes. From an engineering perspective, standardized acquisition protocols are equally critical for interoperability with wearable sensors and multimodal biosignal systems.

Nevertheless, current evidence remains insufficient to justify widespread clinical deployment. The predominance of small, single-center studies with variable reporting underscores the need for standardized methodological frameworks.

Translation of periocular sEMG into clinical decision-support tools will likely require adherence to established regulatory pathways for medical electrical equipment and diagnostic technologies. In the United States, sEMG-based systems intended for diagnostic use would typically require clearance through the U.S. Food and Drug Administration (FDA), most commonly via the 510(k) pathway, which necessitates demonstration of substantial equivalence, technical reliability, and clinical performance. Similarly, international deployment may depend on compliance with standards developed by the International Organization for Standardization (ISO) and the International Electrotechnical Commission (IEC), including requirements for instrument safety, calibration, signal integrity, and risk management. Early alignment of periocular sEMG research with these regulatory expectations—particularly through standardized acquisition protocols and validated analytical pipelines—may help accelerate future clinical translation while reducing barriers to approval.

The framework proposed here should therefore be viewed as enabling infrastructure for future validation rather than as confirmation of immediate translational readiness.

### 4.5. Research Roadmap and Future Directions

Progress toward standardization will require multicenter collaboration, harmonized protocols, shared datasets, and consensus on normalization strategies that do not rely exclusively on maximal voluntary contraction. Reducing crosstalk and aligning acquisition timing with clinical interventions should be considered immediate priorities.

The framework presented here is intended as a starting point for these efforts rather than a definitive standard.

## 5. Conclusions

Periocular sEMG is an evolving but methodologically fragmented field. This review provides a consolidated methodological synthesis, a periocular-specific conceptual framework, and a minimum reporting set to guide future research. Broad adoption and iterative refinement of these principles will be essential before periocular sEMG can be reliably translated into routine clinical and technological practice.

## Figures and Tables

**Table 1:** Summary of Basic Characteristics and Findings of Included sEMG Eyelid Studies.

Study	Diagnosis	N (Patients/Controls)	Age (Mean or Range)	Sex Distribution	Study Design
Gehricke et al. (2002) [28]	Healthy	15 (15/0)	20–40	Not stated	Experimental
VanderWerf et al. (2007) [31]	Bell’s palsy	9 (9/0)	Not stated	Mixed	Longitudinal observational
Richard et al. (2009) [21]	Blepharospasm	14 (7/7)	Not stated	Mixed	Analytical observational (case–control)
Price et al. (2010) [27]	Blepharospasm	7 (7/0)	50–70	Mixed	Pilot
Frigerio et al. (2013) [15]	Healthy	15 (15/0)	26.4 ± 3.1	5 F/5 M	Experimental
Tu et al. (2016) [24]	Ptosis correction	16 (4/12)	65.1 ± 9.2	Mixed	Prospective surgical cohort
Yılmaz et al. (2021) [19]	Hemifacial spasm	30 (30/0)	56.7 ± 11.5	21 F/11M	RCT
Krajewska-Węglewicz et al. (2022) [25]	Ptosis surgery	68 (29/39)	Not stated	Mixed	Prospective Observational
Moon et al. (2023) [22]	Forehead rejuvenation	31 (31/0)	Not stated	All F	Prospective interventional (split-face)
Hollander et al. (2023) [20]	Blepharoplasty	54 (54/0)	45–75	Predominantly F	RCT
Krajewska-Węglewicz et al. (2023) [14]	Dermatochalasis	26 (26/0)	60–75	Mixed	Pilot
Steiner et al. (2024) [26]	Facial palsy	17 (17/0)	Not stated	Mixed	Pilot
Schneider et al. (2025) [23]	Facial synkinesis	36 (36/0)	Not stated	Mixed	Prospective interventional
Krajewska-Węglewicz et al. (2025) [29] *	Healthy-demographic determinants	84	27–87	44 F/40M	Cross-sectional study (predictor analysis)
Krajewska-Węglewicz et al. (2025) [7]	Ptosis	54 (28/26)	45–83	46 F/8M	Prospective observational study
Krajewska-Węglewicz et al. (2025) [30] *	Healthy—normative dataset	84	63.12	44 F/40M	Cross-sectional study (normative)

* Studies 2025 [29] and 2025 [30] were derived from the same healthy cohort but addressed distinct analytical objectives; the cohort was counted once when summarizing total participants.

**Table 2:** Summary of Methodological Characteristics and Findings of Included sEMG Eyelid Studies.

Study (Reference)	Electrode Type, Placement, Inter-electrode Distance	Hardware and Acquisition Settings	Outcome Measures (e.g., RMS, Frequency, Blink Rate)	Main Results and Reported Limitations
Gehricke et al., 2002 [28]	Surface electrodes combined with EOG, inter-electrode distance NR	Sampling rate NR; filtering NR	Temporal and amplitude characteristics of blinks	Differentiated spontaneous vs. reflex blinks; limitations: pediatric study, generalizability
VanderWerf et al., 2007 [31]	Ag/AgCl surface electrodes, OO placement; inter-electrode distance NR	Sampling rate NR; filtering NR	Blink kinematics, blink rate, EMG activity	OO-EMG and blink kinematics characterized during various blink types; limitations: small sample, lack of sampling details
Richard et al., 2009 [21]	Surface electrodes, eyelid position, inter-electrode distance NR	Portable dual-channel EMG recorder; settings NR	Blink rate, muscle activity during reading tasks	Demonstrated EMG changes during reading; limitations: small sample, limited electrode details
Price et al., 2010 [27]	Miniature surface electrodes over eyelid muscles	Sampling rate NR; filtering NR	Blink frequency, EMG signal amplitude	Slight improvements in symptoms after methylphenidate; limitations: pilot study, small sample
Frigerio et al., 2012 [15]	Surface electrodes for detection	Sampling rate NR; filtering NR	Detection of EMG activity for artificial blink pacing	Proposed artificial blink system; limitations: proof-of-concept only
Tu et al., 2016 [24]	Surface electrodes (specifics NR), pretarsal placement	Sampling rate NR; filtering NR	Detrended Fluctuation Analysis (DFA) of EMG signal	Temporal correlations in sEMG signals evaluated pre-/post-surgery; limitations: lack of electrode placement specifics
Yilmaz et al., 2020 [19]	Placement NR	Sampling rate NR; filtering NR	Blink reflex amplitude, latency pre-/post-botulinum toxin	BTX effects evaluated; limitations: incomplete methodological transparency
Moon et al., 2023 [22]	Surface electrodes, forehead (frontalis muscle)	Sampling rate NR; filtering NR	Eyebrow height/movement, EMG activity	Split-face comparison of rejuvenation effects; limitations: forehead focus, not eyelid-centered
Krajewska-Węglewicz et al., 2022 [25]	Mid-pretarsal sEMG electrodes	Sampling rate NR; filtering NR	RMS values of EMG activity pre-/post-surgery	Increased muscle activation post-surgery; limitations: lack of long-term follow-up
Hollander et al., 2023 [20]	Placement NR	Sampling rate NR; filtering NR	EMG amplitude, frequency content, blink reflex	Compared techniques in blepharoplasty; limitations: electrode details incomplete
Krajewska-Węglewicz et al., 2023 [14]	Mid-pretarsal placement, sEMG electrodes	Sampling rate NR; filtering NR	EMG signal strength, muscle ultrastructure analysis	Age-related reduction in OO strength; limitations: small sample, pilot study
Steiner et al., 2024 [26]	Placement NR	Simulation-based, no specific sampling data	EMG input for closed-loop stimulation	Closed-loop simulation; limitations: simulation-only, requires in vivo testing
Schneider et al., 2025 [23]	High-resolution surface electrodes	Sampling rate NR; filtering NR	RMS values pre-/post-training, EMG biofeedback	Demonstrated EMG improvement post-training; limitations: pilot, no control group
Krajewska-Węglewicz et al. (2025) [29] *	Mid-pretarsal placement, sEMG electrodes	Sampling rate NR; filtering NR	RMS-MVC and RMS-GEC values; analysis of age, gender, and laterality effects	Designed to determine demographic influences on OOM sEMG and evaluate bilateral equivalence. Limitations implied: need for further investigation of physiological modifiers
Krajewska-Węglewicz et al. (2025) [7]	Mid-pretarsal placement, sEMG electrodes	Sampling rate NR; filtering NR	Electromyographic patterns of OOM and LPS to evaluate dynamic muscle interplay in ptosis before and after surgery	Designed to characterize neuromuscular mechanisms contributing to inferior scleral show and improve diagnostic insight
Krajewska-Węglewicz et al. (2025) [30] *	Mid-pretarsal placement, sEMG electrodes	Sampling rate NR; filtering NR	Mean amplitude, maximal amplitude, RMS for MVC and GEC; %MVC and GEC/MVC RMS ratio	Established normative periocular sEMG benchmarks. Limitations: need for standardized placement due to sensitivity of amplitude to electrode shifts

**Notes:** NR = Not Reported; Many studies lacked full details of hardware or signal acquisition parameters, limiting reproducibility. Future research should standardize reporting of electrode specifications and acquisition settings in eyelid sEMG studies. * Studies 2025 [29] and 2025 [30] were derived from the same healthy cohort but addressed distinct analytical objectives; the cohort was counted once when summarizing total participants.

**Table 3:** Methodological limitations of included periocular sEMG studies.

Study	Sample Size Limitation	Design Limitation	Reporting Limitation	Additional Concerns
Gehricke 2002 [28]	Very small healthy cohort	Experimental physiology	Limited acquisition detail	Early analog technology
VanderWerf 2007 [31]	Small clinical sample	Observational	Partial electrode description	No normalization
Richard 2009 [21]	Small case–control	Observational	Incomplete preprocessing details	Limited reproducibility
Price 2010 [27]	Pilot sample	Feasibility design	Minimal methodological justification	Exploratory outcomes
Frigerio 2013 [15]	Small healthy cohort	Experimental	Partial acquisition parameters	No scaling strategy
Tu 2016 [24]	Moderate sample	Surgical cohort	Limited electrode mapping	Postoperative timing variability
Yılmaz 2021 [19]	Moderate	RCT	Good reporting	Intervention timing variability
Krajewska-Węglewicz 2022 [25]	Moderate	Prospective observational	Partial normalization description	Single-center
Moon 2023 [22]	Moderate	Split-face	Adequate	Procedure-specific
Hollander 2023 [20]	Moderate–large	RCT	Strong reporting	Population skew (mostly female)
Krajewska-Węglewicz 2023 [14]	Small	Pilot	Limited parameter detail	Exploratory
Steiner 2024 [26]	Small	Pilot	Partial electrode reporting	Clinical heterogeneity
Schneider 2025 [23]	Moderate	Prospective interventional	Good	No consensus epoch strategy
Krajewska-Węglewicz 2025 [29]	Large	Cross-sectional	Strong	No longitudinal validation
Krajewska-Węglewicz 2025 [7]	Moderate	Prospective observational	Strong	Surgical population
Krajewska-Węglewicz 2025 [30]	Large (shared cohort)	Cross-sectional	Strong	Secondary analysis

**Table 4:** Recommended methodological framework for standardized periocular sEMG acquisition and reporting.

Parameter	Recommendation	Rationale/Notes
Electrode Type	Miniaturized Ag/AgCl surface electrodes (<10 mm diameter), suitable for both clinical and wearable systems.	Small recording surfaces improve spatial selectivity for periocular muscles and reduce crosstalk from adjacent facial and extraocular muscles. Compatible with reusable or disposable configurations.
Electrode Placement	Mid-pretarsal placement for OOM specificity; lateral canthal placement for robust blink detection and inter-side comparisons. Maintain fixed inter-electrode spacing of 10–15 mm and clearly document electrode orientation and polarity.	Pretarsal alignment enhances reproducibility and minimizes variability in eyelid closure signals. Lateral placement improves blink detection in dynamic tasks and supports symmetry analysis.
Reference Electrode	Place on the forehead or mastoid process, maintaining consistent positioning across participants and sessions.	Stable reference placement reduces motion artifacts and inter-session variability. Forehead location preferred for ease of access; mastoid placement may improve signal stability in high-motion protocols.
Acquisition Settings	Band-pass filter 20–500 Hz; notch filter 50/60 Hz as appropriate; amplifier gain 1000–2000×; sampling rate ≥ 1000 Hz (≥2000 Hz for high-density arrays).	Ensures adequate bandwidth for periocular motor unit potentials and compatibility with both analog and digital amplifiers. Sampling rates ≥ 2000 Hz recommended for modern digital or wearable systems.
Signal Processing	Apply full-wave rectification; extract RMS or mean absolute value (MAV); perform baseline correction to remove tonic activity. Normalize to maximal voluntary contraction (MVC) when feasible, or to a standardized blink amplitude in non-MVC protocols. Adaptive filtering may be used for ocular artifact suppression.	Enhances cross-study comparability and supports reproducible quantitative analyses. Normalization and adaptive filtering improve signal quality for both physiological interpretation and machine-learning applications.
Outcome Measures	Amplitude-based metrics (RMS, MAV, peak activation); symmetry indices (inter-side ratio, symmetry index); temporal measures (blink frequency, latency, and duration); task-specific activation profiles (voluntary vs. reflexive closure).	Enables multidimensional interpretation of muscle performance, recovery, or intervention effects. Supports integration with automated analytical pipelines and AI-driven feature extraction.
Reporting Standards	Report: (1) electrode type, placement map, and inter-electrode distance; (2) acquisition parameters (filters, gain, sampling rate); (3) normalization and preprocessing procedures; (4) participant state and task design; (5) inclusion/exclusion criteria.	Transparent reporting ensures reproducibility, facilitates meta-analytic comparison, and supports AI-based secondary analyses. A structured checklist should accompany future periocular sEMG studies.
Future Integration	Encourage compatibility with high-density, flexible, or wearable sEMG arrays; promote multimodal integration with EOG, PPG, IMU, and OCT sensors for multimodal facial assessment.	Future-proofing of methodology will enable continuous monitoring, improved clinical diagnostics, and translational applications in rehabilitation, neuroengineering, and human–computer interaction.

## Data Availability

No new data were created or analyzed in this study.

## Abbreviations

The following abbreviations are used in this manuscript:

sEMG	Surface electromyography
OOM	Orbicularis Oculi Muscle
LPS	Levator Palpebrae Superioris
SENIAM	Surface Electromyography for the Non-invasive Assessment of Muscles
PPC	Population–Concept–Context
RMS	Root Mean Square
MAV	Mean Absolute Value

## Appendix A. Preferred Reporting Items for Systematic Reviews and Meta-Analyses Extension for Scoping Reviews (PRISMA-ScR) Checklist

**SECTION**	**ITEM**	**PRISMA-ScR CHECKLIST ITEM**	**REPORTED ON PAGE #**
**TITLE**			
Title	1	Identify the report as a scoping review.	1
**ABSTRACT**			
Structured summary	2	Provide a structured summary that includes (as applicable): background, objectives, eligibility criteria, sources of evidence, charting methods, results, and conclusions that relate to the review questions and objectives.	1–2
**INTRODUCTION**			
Rationale	3	Describe the rationale for the review in the context of what is already known. Explain why the review questions/objectives lend themselves to a scoping review approach.	3
Objectives	4	Provide an explicit statement of the questions and objectives being addressed with reference to their key elements (e.g., population or participants, concepts, and context) or other relevant key elements used to conceptualize the review questions and/or objectives.	3
**METHODS**			
Protocol and registration	5	Indicate whether a review protocol exists; state if and where it can be accessed (e.g., a Web address); and if available, provide registration information, including the registration number.	4
Eligibility criteria	6	Specify characteristics of the sources of evidence used as eligibility criteria (e.g., years considered, language, and publication status), and provide a rationale.	4
Information sources *	7	Describe all information sources in the search (e.g., databases with dates of coverage and contact with authors to identify additional sources), as well as the date the most recent search was executed.	5
Search	8	Present the full electronic search strategy for at least 1 database, including any limits used, such that it could be repeated.	5
Selection of sources of evidence †	9	State the process for selecting sources of evidence (i.e., screening and eligibility) included in the scoping review.	5
Data charting process ‡	10	Describe the methods of charting data from the included sources of evidence (e.g., calibrated forms or forms that have been tested by the team before their use, and whether data charting was done independently or in duplicate) and any processes for obtaining and confirming data from investigators.	6
Data items	11	List and define all variables for which data were sought and any assumptions and simplifications made.	6
Critical appraisal of individual sources of evidence §	12	If done, provide a rationale for conducting a critical appraisal of included sources of evidence; describe the methods used and how this information was used in any data synthesis (if appropriate).	N/A
Synthesis of results	13	Describe the methods of handling and summarizing the data that were charted.	6
**RESULTS**			
Selection of sources of evidence	14	Give numbers of sources of evidence screened, assessed for eligibility, and included in the review, with reasons for exclusions at each stage, ideally using a flow diagram.	7
Characteristics of sources of evidence	15	For each source of evidence, present characteristics for which data were charted and provide the citations.	8–10
Critical appraisal within sources of evidence	16	If done, present data on critical appraisal of included sources of evidence (see item 12).	12, 14
Results of individual sources of evidence	17	For each included source of evidence, present the relevant data that were charted that relate to the review questions and objectives.	15–17
Synthesis of results	18	Summarize and/or present the charting results as they relate to the review questions and objectives.	18
**DISCUSSION**			
Summary of evidence	19	Summarize the main results (including an overview of concepts, themes, and types of evidence available), link to the review questions and objectives, and consider the relevance to key groups.	18–25
Limitations	20	Discuss the limitations of the scoping review process.	21
Conclusions	21	Provide a general interpretation of the results with respect to the review questions and objectives, as well as potential implications and/or next steps.	26
**FUNDING**			
Funding	22	Describe sources of funding for the included sources of evidence, as well as sources of funding for the scoping review. Describe the role of the funders of the scoping review.	26

JBI = Joanna Briggs Institute; PRISMA-ScR = Preferred Reporting Items for Systematic reviews and Meta-Analyses extension for Scoping Reviews. * Where *sources of evidence* (see second footnote) are compiled from, such as bibliographic databases, social media platforms, and Web sites. † A more inclusive/heterogeneous term used to account for the different types of evidence or data sources (e.g., quantitative and/or qualitative research, expert opinion, and policy documents) that may be eligible in a scoping review as opposed to only studies. This is not to be confused with *information sources* (see first footnote). ‡ The frameworks by Arksey and O’Malley (6) and Levac and colleagues (7) and the JBI guidance (4, 5) refer to the process of data extraction in a scoping review as data charting. § The process of systematically examining research evidence to assess its validity, results, and relevance before using it to inform a decision. This term is used for items 12 and 19 instead of ‘risk of bias’ (which is more applicable to systematic reviews of interventions) to include and acknowledge the various sources of evidence that may be used in a scoping review (e.g., quantitative and/or qualitative research, expert opinion, and policy document). *From:* Tricco AC, Lillie E, Zarin W, O’Brien KK, Colquhoun H, Levac D, et al. PRISMA Extension for Scoping Reviews (PRISMAScR): Checklist and Explanation. Ann Intern Med. 2018;169:467–473. doi: 10.7326/M18-0850 [16].

## Appendix B

Full Search Strategies

Database: PubMed (MEDLINE)

1. “Electromyography”[Mesh]
2. “Electromyography”[tiab]
3. “surface electromyography”[tiab]
4. sEMG[tiab]
5. #1 OR #2 OR #3 OR #4
6. Orbicularis Oculi Muscle[Mesh]
7. orbicularis oculi[tiab]
8. levator palpebrae superioris[tiab]
9. eyelid muscle[tiab]
10. #6 OR #7 OR #8 OR #9
11. “Eyelids”[Mesh]
12. eyelid*[tiab]
13. periocular[tiab]
14. #11 OR #12 OR #13
15. #5 AND #10 AND #14

Database: Embase (Elsevier)

1. ‘electromyography’/exp
2. ‘electromyography’:ti,ab
3. ‘surface electromyography’:ti,ab
4. semg:ti,ab
5. #1 OR #2 OR #3 OR #4
6. ‘orbicularis oculi muscle’/exp
7. ‘orbicularis oculi’:ti,ab
8. ‘levator palpebrae superioris’:ti,ab
9. ‘eyelid muscle’:ti,ab
10. #6 OR #7 OR #8 OR #9
11. ‘eyelid’/exp
12. eyelid*:ti,ab
13. periocular:ti,ab
14. #11 OR #12 OR #13
15. #5 AND #10 AND #14

Database: Web of Science Core Collection (Clarivate)

TS = (“electromyography” OR “surface electromyography” OR sEMG)

AND TS = (“orbicularis oculi” OR “levator palpebrae superioris” OR “eyelid muscle”)

AND TS = (eyelid* OR periocular)
